# Systematic profiling of SARS-CoV-2 structural protein-specific T cell epitopes in Omicron infections following inactivated vaccination

**DOI:** 10.1016/j.isci.2026.114891

**Published:** 2026-02-03

**Authors:** Zhiqing Li, Mengmeng Cui, Jian Wu, Tianju Hu, Junyan Dan, Xiaosu Chen, Qicong Shen, Jin Hou, Zhongfang Wang, Yizhi Yu, Shuxun Liu

**Affiliations:** 1 National Key Laboratory of Immunity and Inflammation, Institute of Immunology, Naval Medical University, Shanghai 200433, China; 2 Department of Immunology, Center for Immunotherapy, Chinese Academy of Medical Sciences, Beijing 100005, China; 3 Laboratory of Advanced Biotechnology, Beijing Institute of Biotechnology, Beijing 100071, China; 4 Frontier Research Center for Cell Response, Institute of Immunology, College of Life Sciences, Nankai University, Tianjin 300071, China; 5 Guangzhou Laboratory, Guangzhou 510300, China

**Keywords:** molecular biology, immunology, virology

## Abstract

Comprehensive identification of T cell epitopes is crucial for understanding SARS-CoV-2-specific T cell immunity and guiding vaccine development. Epitope-specific T cells primed by inactivated vaccination (IV)—widely administered in China—followed by Omicron breakthrough infection (BI)—remain poorly characterized. Using hierarchical epitope screening in the IV-BI cohort, we systematically validated known epitope-HLA interactions and uncovered previously unrecognized associations, revealing immunodominant epitopes in conserved regions (S1-NTD, M protein β-sandwich, and N-CTD). Based on these epitopes, analysis of cohort samples before and after BI revealed that certain epitope-specific, CD4^+^ T cells were initially primed by IV. Longitudinal analysis revealed epitope-specific temporal dynamics in SARS-CoV-2-specific T cell immunity, with certain subsets declining over 10 months post-infection. While XBB.1.5 and BA.2.86 subvariant-harboring mutations partially evaded T cell immunity, a conservative HLA-DRB1∗09:01-restricted epitope was identified across ancestral and Omicron strains. These findings delineate regionally prevalent HLA-associated immunodominant epitope frameworks and highlight pan-variant epitope candidates for vaccine development and T cell immunity monitoring.

## Highlights

• Characterizing dominant HLA-epitope pairs by inactive vaccine and Omicron infection
• Inactive vaccination elicits broad-spectrum CD4^+^ T but not CD8^+^ T cell response
• Mutated S_152–160_, S_360–370_, and S_126–140_ in XBB.1/BA2.86 variant impair T cell recognition
• SARS-CoV-2 T cell clones show varying durability based on epitopes

## Graphical abstract

**Figure:**
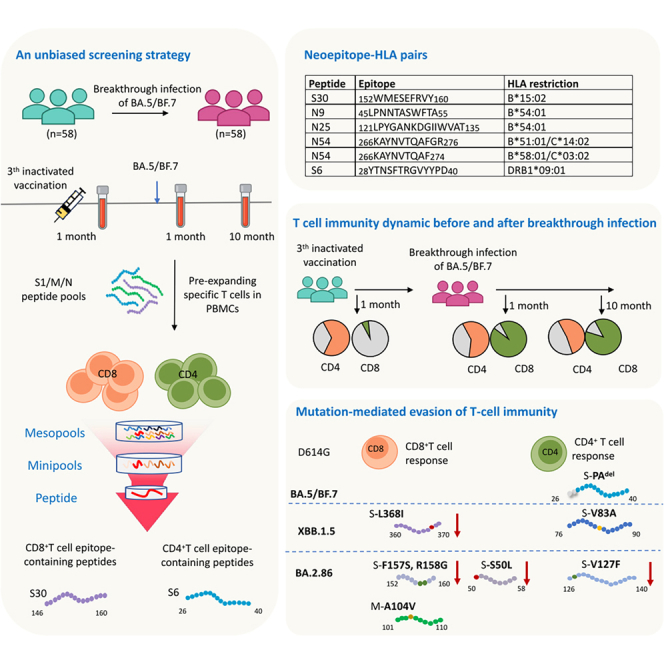

## Introduction

The COVID-19 pandemic, caused by SARS-CoV-2, remains a major global health challenge, with enduring public health implications. In China, widespread administration of three-dose inactivated vaccines significantly reduced COVID-19 severity and mortality but was also challenged by subsequent emergence of antigenically divergent Omicron subvariants. These mutations drastically compromised neutralizing capacity in vaccinated individuals, leading to breakthrough infections, even under large vaccination coverage. Although COVID-19 pandemic has now entered an endemic phase, the persistent evolution of variants of concern (VOCs) underscores an enduring threat of immune evasion and viral resurgence.^1^^,^^2^ Therefore, a comprehensive understanding of SARS-CoV-2-specific adaptive immunity, particularly T cell immunity, is critical for the development of next-generation vaccines capable of inducing pan-variant immunity.

Unlike B cell responses primarily targeting the highly mutable spike protein, T cell responses recognize evolutionarily constrained epitopes spanning multiple structural (e.g., nucleocapsid and membrane) and non-structural SARS-CoV-2 antigens, enabling broader cross-reactivity against viral variants. This multi-antigen targeting strategy inherently limits immune escape by VOCs, as VOC evolution remains largely confined to the spike protein. Deciphering epitope-specific T cells is critical for precisely quantifying the magnitude, kinetics, and phenotypic profiles of SARS-CoV-2-specific T cell immunity, thereby establishing a comprehensive immunological framework to guide vaccine design.

To date, the immune epitope database (IEDB) has registered over 2,000 SARS-CoV-2-specific T cell epitopes, encompassing both experimentally validated epitopes and a larger number of unvalidated, computationally predicted candidates.^3^^,^^4^^,^^5^^,^^6^^,^^7^ However, the human leukocyte antigen (HLA) system exhibits substantial ethnic and geographical diversity, implying that numerous region-specific and ethnic HLA-associated epitopes remain undiscovered. Systematic profiling of region- and ethnicity-specific, HLA-associated epitopes enables the development of pan-variant T cell-based vaccines and T cell immunity monitoring. To date, most identified T cell epitope-HLA pairs have been derived from mRNA-vaccinated Western populations, whereas immunodominant epitopes relevant to Chinese cohorts primarily immunized with inactivated vaccines remain poorly characterized.^8^^,^^9^

Current approaches for profiling T cell epitopes rely predominantly on combining computational prediction of epitope-HLA interactions with experimental validation using overlapping peptide pools covering target antigens.^10^^,^^11^^,^^12^ In addition, recent advances in high-throughput functional screening have enabled robust epitope discovery, including T-Scan systems for identifying CD8^+^ T cell epitopes, as well as MHC-associated peptide proteomics combined with mass spectrometry and mammalian epitope display systems for CD4^+^ T cell epitopes.^13^^,^^14^^,^^15^

In this study, we implemented an unbiased screening strategy employing hierarchical peptide pools (mesopools, minipools, and single peptide) to systematically identify SARS-CoV-2 structural protein-specific T cell epitopes in Omicron BA.5 or BF.7 breakthrough infection following inactivated vaccination cohorts. Our study systematically validated previously reported epitope-HLA interactions and identified previously unrecognized associations, delineating regionally prevalent HLA-associated immunodominant epitope frameworks and their structural location preferences. Based on epitope-specific T cell responses, longitudinal analysis confirmed the pre-existence of SARS-CoV-2-specific CD4^+^ T cells initially primed by inactivated vaccination and revealed epitope-specific temporal dynamics of T cell immunity post-breakthrough infection. Despite partial immune evasion of later emerging Omicron subvariants, XBB.1.5 and BA.2.86, from established T cell immunity, a conservative DRB1∗09:01-restricted epitope was identified across ancestral and Omicron strains, providing pan-variant epitope candidate for vaccine development and T cell immunity monitoring.

## Results

### Identification of immunodominant T cell epitope-containing peptides in SARS-CoV-2 structural proteins S1, M, and N

To characterize the immunodominance and breadth of T cell epitope patterns induced by SARS-CoV-2 inactivated vaccination followed by Omicron BA.5 or BF.7 breakthrough infection, we analyzed peripheral blood mononuclear cell (PBMC) samples from 58 volunteers. All participants had received three doses of inactivated vaccines prior to experiencing Omicron BA.5 or BF.7 breakthrough infection, with clinical outcomes categorized as mild disease. The demographic characteristics of these volunteers are detailed in Table S1.

**Figure 1:**
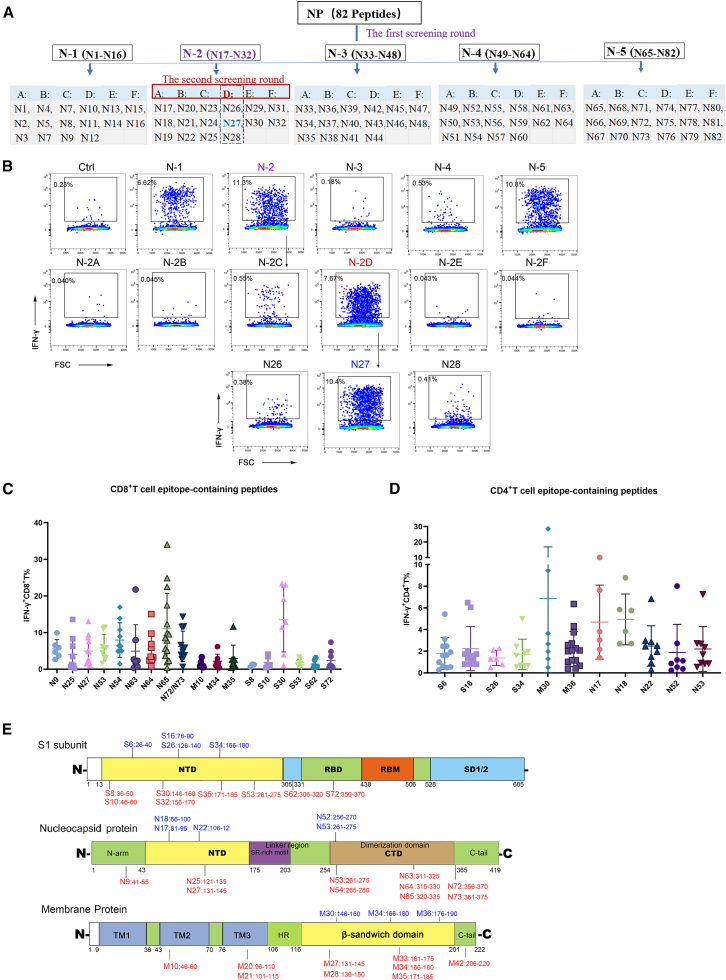
Identification of T cell epitope-containing peptides within S1, M, and N proteins (A) Screening strategy for the identification of T cell epitope-containing peptides within the SARS-CoV-2 N protein, using hierarchical peptide pools. (B) FACS plots showing one representative screening workflow for identifying the N27 peptide, which contains a CD8^+^ T cell epitope, after three rounds of screening. (C and D) IFN-γ^+^ T cell frequencies in total CD8^+^ T cells (C) or CD4^+^ T cells (D), measured after initially priming with S1/M/N peptide pools for 9 days, followed by restimulation with cognate 15-mer peptides from BA.5/BF.7 convalescents. Data are calculated by subtracting the DMSO group from the peptide-stimulating group. Data represent independent biological replicates, shown as the mean ± SEM. (E) Distribution of T cell epitope-containing peptides within SARS-CoV-2 S1, N, and M proteins. CD8^+^ T cell epitopes are denoted in red, and CD4^+^ T cell epitopes are denoted in blue. FACS, Fluorescence-activated cell sorting.

We initially stimulated unexpanded PBMCs with the S1 peptide pool for 16 h and observed an extremely low frequency of antigen-specific T cells, which hindered the clear identification of immunodominant epitopes (Figures S1A–S1C). To address this limitation, we expanded antigen-reactive T cells by stimulating PBMCs with the S1, N, or M peptide libraries, respectively. This approach effectively expanded antigen-specific T cells (Figures S1D and S1E), which subsequently secreted IFN-γ and TNF-α. Among these, CD8^+^ T cells expressed granzyme B (GZMB), while CD4^+^ T cells expressed PD-1 (Figures S1F and S1G). Based on these findings, we implemented a hierarchical peptide pool screening strategy—using mesopools, minipools, and individual peptides—to identify T cell epitopes specific to SARS-CoV-2 structural proteins in expanded PBMCs. Epitope-containing peptides from the S1 and N pools were identified through three rounds of iterative screening, whereas those from the M pool required only two rounds of screening due to its smaller peptide library size (Figures 1A and S2A–S2D).

To illustrate the screening workflow, we describe the identification of the N27 peptide, which harbors a CD8^+^ T cell epitope. The N peptide pool-expanded PBMCs were distributed across dozens of wells for sequential stimulation with mesopools, minipools, and finally, single peptides. Initial screening with five N mesopools identified *N*-1, *N*-2, and *N*-5 as immunodominant, which were advanced to minipool screening. As shown for *N*-2 (Figure 1B), the N-2D minipool (N26–N28) was subsequently pinpointed as being immunodominant. Finally, single peptide screening demonstrated that the peptide N27 induced significantly stronger CD8^+^ T cell activation than N26 and N28, confirming its epitope-bearing properties (Figure 1B).

Through the screening approach as above, we identified 27 CD8^+^ T cell epitopes, comprising 8, 9, and 10 peptides from the S1, M, and N proteins, respectively (Table S2). Nineteen of these peptides elicited CD8^+^ T responses in at least three of the 58 participants (Figure 1C). For CD4^+^ T cell epitope-containing peptides, 12 peptides were identified (4 S1-derived, 3 M-derived, and 5 N-derived), with 11 peptides activating CD4^+^ T cells in at least three participants (Figure 1D and Table S3). These epitope-containing peptides were mainly located in the N-terminal domain (NTD) of S1 subunit, both the NTD and C-terminal domain (CTD) of N protein, and the β-sandwich domain of M protein (Figure 1E), which harbors multiple overlapping CD4^+^/CD8^+^ T cell epitopes that have been reported in prior studies.^16^^,^^17^^,^^18^

### Identification of SARS-CoV-2-specific CD8^+^ T cell epitopes and their corresponding HLA restrictions

To determine CD8^+^ T cell epitopes and their MHC-I restrictions, we performed HLA allele sequencing for all participants (Table S2) and employed NetMHCpan 4.1 to systematically evaluate the binding affinity of candidate epitopes to responder’s HLA class I alleles, using a binding threshold of <2% rank.^13^ We predicted epitopes within peptides and their HLA restrictions through a combination of binding threshold and overlapping HLA I alleles of responders, which are summarized in Table 1.

**Table 1:** Predicted CD8^+^ T cell epitopes and HLA restriction by NetMHCpan 4.1

Peptide ID	IEDB	Predicted epitope sequence	Predicted HLA restrictions	Rank_EL (%)	Participants who responded	Response rate
**S10**	**#N/A**	_46_***SVLHSTQDL***_54_FLPFFS	C∗01:02	1.211	I-13	1/16
	1333812	SVLH_50_***STQDLFLPF***_58_FS	A∗24:02	1.100	I-7 and I-77	2/16
			B∗15:01	0.767	I-65	1/6
			C∗12:02	0.583	I-66 and I-389	2/2
			B∗13:01	1.332	I-389	1/10
**S30**	2134346	HKNNKS_152_***WMESEFRVY***_160_	**B∗15:02**^**N**^	0.217	I-229, I-62, I-54, I-73, I-34, and I-77	**6/6**
			B∗15:27	0.381	I-62	1/1
			B∗15:25	0.242	I-25	1/1
S53	1711597	GAAA_265_***YYVGYLQPR***_273_TF	A∗33:03	0.177	I-81, I-82, I-20, I-25, and I-85	5/11
	1334182	GAAAYY_267_***VGYLQPRTF***_275_	C∗07:02	0.476	I-46	1/15
S72	7247	KRISN_361_***CVADYSVLY***_369_^N^	B∗15:02	0.580	I-34, I-77, and I-62	3/6
			A∗11:01	0.557	I-62, I-65, and I-77	3/25
			B∗35:01	0.371	I-178	1/4
			B∗15:01	0.854	I-65	1/8
			B∗46:01	0.674	I-69	1/13
			A∗26:01	0.018	I-14	1/2
M10	1679107	_46_***LYIIKLIFL***_54_WLLWPV	A∗24:02	0.171	I-212, I-47, I-71, I-77, and I-7	5/17
			C∗07:02	0.625	I-72, I-212, I-71, and I-7	4/15
M34/M35	5150	_171_***ATSRTLSYY***K_180_	A∗11:01	0.078	I-389, I-65, I-54, I-7, I-125, I-77, I-149, and I-13	8/25
**N9**	**#N/A**	RPQG_45_***LPNNTASWFTA***_55_	**B∗54:01**^**N**^	0.261	I-145, I-175, I-502, and I-31	**4/5**
	1324011	_41_***RPQGLPNNTA***_50_SWFTA	**B∗55:02**^**N**^	0.087	I-77 and I-13	**2/2**
**N25**	1321105	_121_***LPYGANKDGI***_130_IWVAT	**B∗54:01**^**N**^	1.664	I-31, I-145, and I-502	**3/5**
			B∗51:01	0.627	I-65	1/3
	**#N/A**	LPYGA_126_***NKDGIIWVA***_134_T	**B∗39:01**^**N**^	0.618	I-71 and I-72	**2/2**
N27	4936	IWV_134_***ATEGALNTPK***_143_DH	A∗11:01	0.225	I-389, I-46, I-54, I-62, I-65, I-66, I-502, I-77, I-79, and I-7	10/25
**N53/54**	1074947	_266_***KAYNVTQAF***_274_G	B∗27:04	1.336	I-389 and I-66	2/2
			B∗13:01	0.122	I-81 and I-389	2/10
			B∗46:01	0.004	I-149	1/13
			**B∗58:01**^**N**^	0.077	I-20, I-81, I-82, I-25, and I-366	**5/8**
**N54**	**#N/A**	_266_***KAYNVTQAFGR***_276_RGPE	A∗11:01	1.438	I-65, I-66, I-366, and I-389	4/25
N63	4307	_311_***ASAFFGMSR***_319_IGMEVT	A∗11:01	0.100	I-389, I-65, and I-7	3/25
	1542371	A_312_***SAFFGMSRI***_320_GMEVT	B∗51:01	0.257	I-65 and I-70	2/3
N63/64	21347	_316_***GMSRIGMEV***_324_T	A∗02:03	0.199	I-14 and I-51	2/4
			B∗13:02	0.171	I-37 and I-80	2/4
N65	71461	GMEV_325_***TPSGTWLTY***_333_TG	B∗35:01	0.012	I-36, I-178, I-145, and I-125	4/4
			B∗35:02	0.026	I-14	1/1
	190494	G_322_***MEVTPSGTWL***_330_TYTG	B∗40:01	0.264	I-184, I-502, I-366, I-20, I-46, I-51, and I-125	7/8
			B∗40:02	0.942	I-37	1/3
			B∗44:03	0.032	I-85, I-353, and I-31	3/3
N72/73	33667	_361_***KTFPPTEPK***_369_K	A∗11:01	0.006	I-65, I-66, I-62, I-46, I-79, I-228, I-229, I-366, I-389, I-502, and I-77	11/25
			A∗30:01	0.004	I-37 and I-229	2/3

Predicted epitopes within peptides are shown in bold and italic type. ^N^, newly identified HLA restriction; #N/A, not available; response rate, (responsive participants)/(all participants sharing a given HLA). Bold, epitopes in peptides, newly identified HLA restriction, and their corresponding response rate.

Consistent with prior studies, the following epitopes and their corresponding HLA alleles were functionally validated by antigen-specific CD8^+^ T cell responses: (1) the N65 peptide, harboring B∗40:01-restricted N_322–330_ and B∗35:01-restricted N_325–333_ epitopes^10^^,^^12^; (2) the M35 peptide, containing the A∗11:01- and A∗01:01-restricted epitope M_171–180_^13^; and (3) the N27, N63, and N72 peptides, each bearing A∗11:01-restricted epitopes (N_134–143_, N_311–319_, and N_361–369_, respectively).^10^^,^^19^^,^^20^ Notably, predictive mapping identified four previously unrecognized CD8^+^ T cell epitopes (designated #NA pending IEDB assignment) and their HLA-restricting alleles: S_46–54_ (C∗01:02) in S10, N_45–55_ (B∗54:01) in N9, N_126–134_ (B∗39:01) in N25, and N_266–276_ (A∗11:01) in N54. Importantly, we uncovered new restricting HLA alleles for four established epitopes, including B∗15:02 for S_152–160_ (S30), B∗55:02 for N_41–50_ (N9), B∗54:01 for N_121–130_ (N25), and B∗58:01 for N_266–274_ (N53/54) (Table 1).

**Figure 2:**
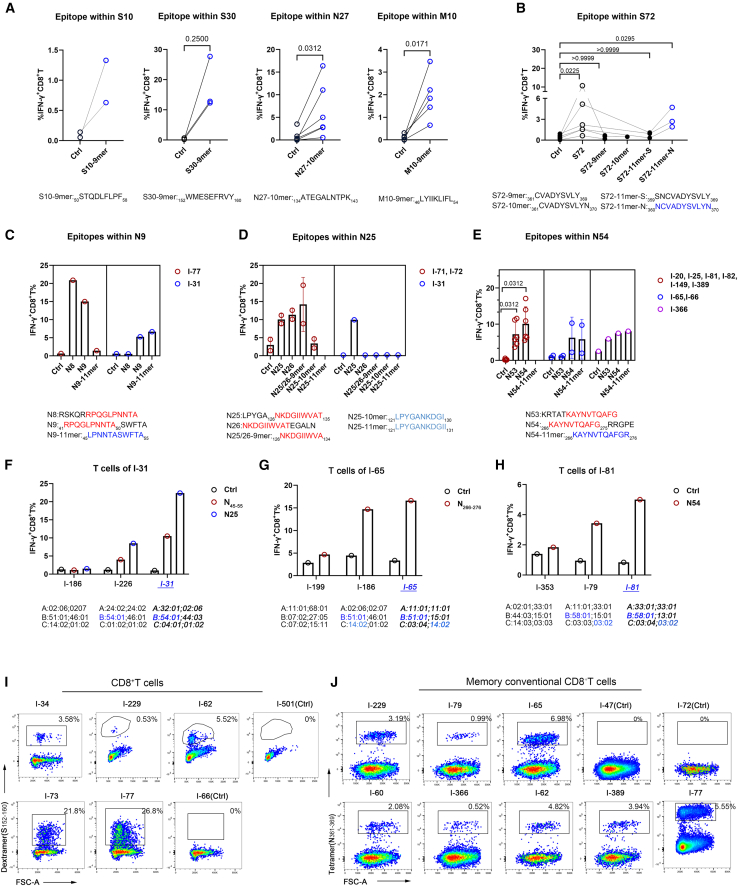
Definition of SARS-CoV-2-specific CD8^+^ T cell epitopes and their HLA-I restrictions (A) Quantification of IFN-γ^+^ CD8^+^ T cell frequencies in total CD8^+^ T cells from S30, S10, M10, and N27 responders by flow cytometry, following stimulation with 9- or 10-mer epitopes or DMSO control. Paired *t* test or Wilcoxon test for data passing or not passing normality tests, respectively. (B) Flow cytometry detection of IFN-γ^+^ CD8^+^ T cell frequencies in PBMCs from S72 responders following stimulation with indicated 9- to 11-mer epitopes within the S72 peptide. Kruskal-Wallis test. (C–E) Flow cytometry detection of IFN-γ^+^ CD8^+^ T cell frequencies of N peptide pool-pre-expanded PBMCs from N54, N9, and N27 responders, following stimulation with 9- to 11-mer epitopes. Wilcoxon test in (E). (F–H) Flow cytometry detection of IFN-γ^+^ CD8^+^ T cell frequencies of N peptide pool-pre-expanded PBMCs from N9 (I-31), N25 (I-31), and N54 (I-65 and I-81) responders, following stimulation with allogeneic PBMCs pulsed with the N9, N25, or N54 peptides, respectively, from donors with partial HLA class I allele matching. Control (Ctrl), DMSO. HLA-I alleles of participants are shown, with common allele highlighted. (I) Flow cytometry plots showing the S_152-160_-B∗15:02 dextramer^+^ cells in gated CD8^+^ T cells in S1 peptide pool-expanded PBMCs from representative responders and non-responder controls (Ctrl, I-501, and I-66). (J) Flow cytometry plots showing the N_361-369_-A∗11:01 tetramer^+^ cells gated on memory conventional CD8^+^ T cells in N peptide pool-expanded PBMCs from representative responders and non-responder controls (Ctrl, I-47, and I-72). The gating strategy for memory conventional CD8^+^ T cells is shown in Figure S3A. Data represent independent biological replicates (A, B, and D, red circle; E, red and blue circles), with the mean ± SEM shown in D (red circle) and E (red and blue circles). Data represent one of two independent technical replicates using PBMCs from the indicated donors (C–H) and either a single independent experiment or one of two independent experiments using PBMCs from the indicated donors (I and J). PBMCs, peripheral blood mononuclear cells.

To experimentally test our in-silico predictions, we synthesized the corresponding 9–11-mer peptides from candidate longer peptides (S30, N9, N25, N27, N54, M10, S10, and S72) and functionally assessed their capacity to stimulate CD8^+^ T cell responses, using intracellular cytokine staining (ICS) assays. Most predicted epitopes, including S_50–58_ (in S10), S_152–160_ (in S30), N_134–143_ (in N27), and M_46–54_ (in M10), elicited robust CD8^+^ T cell responses (Figure 2A), confirming their identity as epitopes. However, within the S72 peptide, it was the 11-mer epitope (S_360–370_), not the predicted 9-mer peptide (S_361–369_), that induced CD8^+^ T cell response (Figure 2B). This may indicate that the presence of an asparagine (N) residue at both the N and C termini of the epitope is critical for its function.

Three peptides (N9, N25, and N54) were each identified as containing two distinct HLA-epitope pairs (Table 1). We functionally validated specific epitopes within them: N_41–50_ and N_45–55_ (N9); N_126–134_ (N25); and N_266–275_ and N_266–276_ (N54) (Figures 2C–2E). A notable result was found in N25—while the full 15-mer peptide activated CD8^+^ T cells in donor I-31 (B∗54:01), the predicted core epitope (N_121–130_) and other peptides failed to do so (Figure 2D). This discrepancy indicates a limitation in the prediction algorithm for B∗54:01 and suggests that the second epitope in N25 is yet to be identified.

To delineate HLA restriction, we assessed CD8^+^ T cell responses using peptide-pulsed PBMCs from donors with partial HLA-I allele matching. Responses from donor I-31 to the N_45–55_ and N25 peptides were evaluated in PBMCs from partially matched donors I-186 and I-226, which confirmed B∗54:01 as the restricting allele for the N_45–55_ epitope (in N9) and for the unidentified epitope within N25 (Figure 2F). We determined that the restricting allele for the N_266–276_ epitope (in N54) was not A∗11:01 but likely either B∗51:01 or C∗14:02, two alleles in strong linkage disequilibrium (Figure 2G). Finally, we identified B∗58:01 as the restricting allele for the epitope N_266–274_ within the N53/N54 peptides (Figure 2H).

Finally, we validated some epitope-HLA interactions using peptide-MHC (pMHC) tetramer or dextramer staining. Specific CD8^+^ T cells binding to the S_152–160_-B∗15:02 dextramer and memory conventional CD8^+^ T cells specific to the N_361–369_-A∗11:01 tetramer were detectable exclusively in their respective responders but not in non-responders (Figures 2I, 2J, and S3A), and no such reactivity was observed in an irrelevant negative control (Figure S3B). Furthermore, we employed N_361–369_-A∗11:01 tetramer for direct *ex vivo* assessment of antigen-specific CD8^+^ T cells in unexpanded PBMCs; tetramer-specific CD8^+^ T cells could be detectable, albeit at low baseline frequencies (Figure S3C). We also assessed the expression of memory, differentiation, and exhaustion markers on S_152–160_-dextramer^+^CD8^+^ T cells from the donor I-54. A portion of these antigen-specific cells displayed a CD45RA^+^CCR7^−^CD27^−^CD28^−^ phenotype, identifying them as terminally differentiated effector memory T cells (T_EMRA_) (Figure S3D).

### Identification of SARS-CoV-2-specific CD4^+^ T cell epitopes and their corresponding HLA class II restrictions

**Figure 3:**
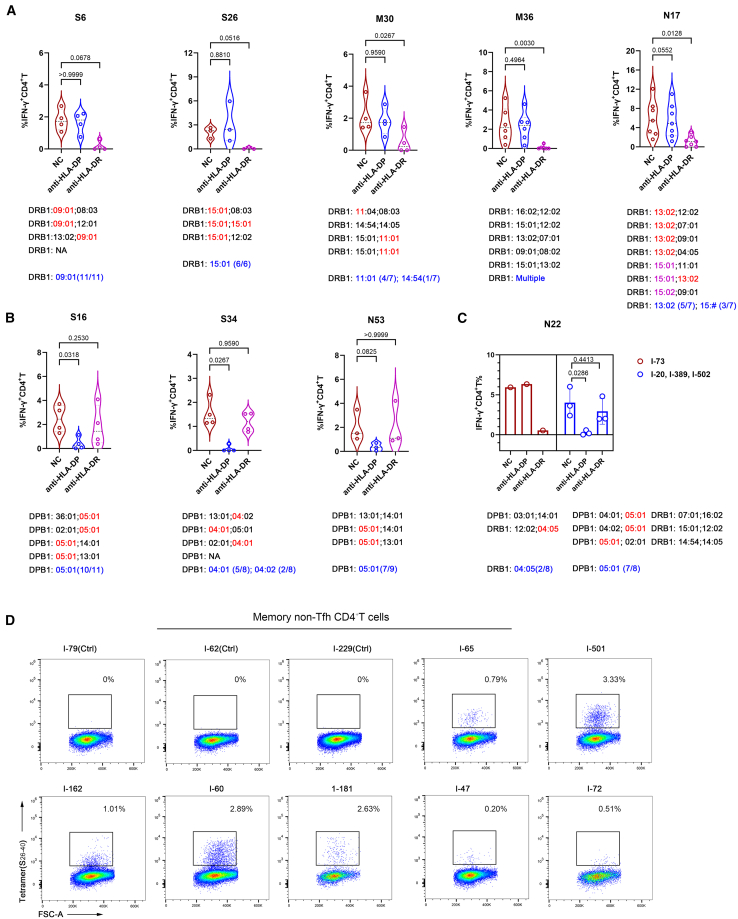
Definition of SARS-CoV-2-specific CD4^+^ T cell epitopes and their corresponding HLA-II restrictions (A) Flow cytometry detection of CD4^+^ T cell responses in S1, N, or M peptide pool-expanded PBMCs from the responders S6, S26, N17, M30, and M36, following stimulation with corresponding peptides, respectively, with or without blockade of anti-HLA-DR or anti-HLA-DP. Common HLA-II alleles of participants in Ab-blocking assays are highlighted in red and their frequencies in all responders in blue. NC, isotype Ig. (B and C) Flow cytometry detection of CD4^+^ T cell responses in S1 peptide pool-pre-expanded PBMCs (B) and N peptide pool-pre-expanded PBMCs (C) from responders, following stimulation with corresponding peptides, respectively, with or without blockade of anti-HLA-DR or anti-HLA-DP. Common HLA-II alleles of participants in Ab-blocking assays are highlighted in red and their frequencies in all responders in blue. (D) Flow cytometry plots showing S26-40-DRB1∗09:01 tetramer^+^ memory non-Tfh CD4^+^ T cells in S1 peptide pool-expanded PBMCs from responders and non-responders. I-79, I-62, and I-229 are non-responding controls. The gating strategy for memory non-Tfh CD4^+^ T cells is shown in Figure S4A. One-way ANOVA or Friedman test for normally or non-normally distributed data, respectively, is shown in (A)–(C). Data represent independent biological replicates (A–C, blue circle). Violin plots display quartile values, and error bar is the mean ± SEM (C, blue circle). Data represent either a single independent experiment or one of two independent experiments using PBMCs from the indicated donors (C, I-73; D). PBMCs, peripheral blood mononuclear cells. NA, not available.

To define the CD4^+^ T cell epitopes, we predicted optimal epitopes within these peptides and their corresponding HLA class II allele in responders, using NetMHCIIpan 4.0, applying a consensus percentile score of <20 as the binding threshold for most epitope-HLA II pairs.^21^ While several epitope-containing peptides aligned with prior reports,^6^^,^^12^^,^^15^^,^^21^ we identified some previously unrecognized binding interactions with alternative HLA class II molecules (Table 2). To validate MHC-II restriction, neutralizing antibodies (Abs) targeting HLA-DR and HLA-DP were introduced during CD4^+^ T cell stimulation. Strikingly, HLA-DR blockade strongly inhibited CD4^+^ T cell responses to S6, S26, M30, M36, and N17/18 peptides, demonstrating an HLA-DR-restricted presentation (Figure 3A). Similarly, HLA-DP blockade abrogated responses to S16, N53, and S34 peptides, validating an HLA-DP-restricted presentation (Figure 3B). Intriguingly, blockade of either HLA-DP or HLA-DR suppressed N22-specific responses in participants carrying DPB1∗05:01 or DRB1∗04:05, respectively (Figure 3C). We excluded DRB1∗12:01 as the MHC class II restriction element for the N22 epitope, as HLA-DR blockade had no effect on the CD4^+^ T cell response of a responder carrying this allele. Thus, N22 comprised two epitopes: one restricted by DPB1∗05:01 and another restricted likely by DRB1∗04:05.

**Table 2:** Predicted CD4^+^ T cell epitopes and corresponding HLA restrictions by NetMHCIIpan 4.0

Peptide ID	IEDB	HLA block	Predicted HLA restrictions	Predicted epitope sequence	Rank_EL	Participants who responded	Response rate
**S6**	1310705	DR	**DRB∗09:01**^**N**^	_26_PAYTNSFTRGVYYPD_40_	3.72	I-7, I-46, I-47, I-60, I-65, I-72, I-85, I-145, I-162, I-181, and I-226	**11/18**
S16	1310848	DP	DPA1∗04:01-DPB1∗05:01	_76_TKRFDNPVLPFNDG_89_	4.7	I-50, I-51, I-54, I-72, I-81, I-85, I-175, I-186, I-212, and I-226	10/41
			DPA1∗03:02-DPB1∗05:01		4.97		
			DQA1∗01:02-DQB1∗03:01	_76_TKRFDNPVLPFN_87_	9.7	I-366, I-51, I-54, I-72, and I-81	5/27
**S26**	1310930	DR	**DRB∗15:01**^**N**^	_126_VVIKVCEFQFCNDPF_140_	78.92	I-37, I-389, I-502, I-47, I-178, and I-71	**6/15**
			DPA1∗01:03-DPB1∗02:01		14.3	I-37, I-178, and I-502	3/20
**S34**	1972691	DP	DPA1∗02:01-DPB1∗04:01	_166_CTFEYVSQPFLMDLE_180_	0.12	I-20, I-43, I-58, I-69, and I-85	5/9
			DPA1∗03:01-**DPB1∗04:02**^**N**^		0.24	I-14 and I-34	**2/3**
			DPA1∗01:03-DPB1∗02:01		1.4	I-181, I-69, and I-34	3/20
M30	1323796	DR	DRB1∗11:01	_146_RGHLRIAGHHLGRCD_160_	0.92	I-61, I-366, I-502, and I-125	4/8
			DRB1∗14:54		3.09	I-212	1/1
M34	1310529	NA	DRB1∗09:01	_166_KEITVATSRTLSYYK_180_	2.06	I-46 and I-181	2/18
**M36**	1310622	DR	**DRB∗16:02**^**N**^	_176_LSYYKLGASQRVAGD_190_	0.83	I-71, I-51, and I-20	**3/4**
			DRB∗07:01		0.81	I-20, I-149, and I-82	3/8
			DRB∗09:01		0.13	I-46, I-65, and I-83	3/18
			DRB∗01:01		0.7	I-13 and I-66	2/2
			DRB∗03:01	_181_LGASQRVAG_189_	8.8	I-81	1/5
			DRB∗15:01	_178_YKLGASQRV_187_	11	I-389 and I-71	2/15
N17/18	1131160	DR	DRB1∗13:02	_86_YYRRATRRIR_95_	0.56	I-25, I-82, I-85, I-175, and I-353	5/5
	1312636		DRB1∗15:01		4.92	I-125 and I-353	2/15
**N22**	1163444	DP	**DPA1∗01:06-DPB1∗05:01**^**N**^	_106_PRWYFYYLGTGPEAG_120_	21.87	I-20, I-51, I-65, I-85, I-175, I-389, and I-502	**7/41**
		DR	DRB1∗04:05		0.66	I-175 and I-73	2/4
**N53**	1312898	DP	**DPA1∗04:01-DPB1∗05:01**^**N**^	_261_KRTATKAYNVTQAFG_275_	3.85	I-20, I-47, I-81, I-85, I-149, I-212, and I-389	**7/41**
			**DPA1∗01:03-DPB1∗03:01**^**N**^		3.6	I-181	**1/3**

^N^, newly identified HLA restriction; NA, not available; response rate, the number of responsive donors/total number of donors carrying a specific HLA. Bold, newly identified HLA restriction and their response rate.

Additionally, we observed discrepancies between computational predictions and experimental data regarding HLA class II restrictions. Specifically, all S16 responders carried DPA1∗02:02, whereas predicted restriction alleles (DPA1∗04:01 and DPA1∗03:02) were absent (Table S4). Additionally, all S26 responders harbored HLA-DRB1∗15:01 despite its computationally predicted weak binding affinity (Figure 3A and Table 2).

Given the high prevalence of DRB1∗09:01 in Han Chinese populations, we synthesized an S_26–40_-DRB1∗09:01 tetramer. Tetramer staining revealed high frequencies of S6-specific memory, non-T follicular helper (Tfh) CD4^+^ T cells in S1 peptide pool-expanded PBMCs, definitively confirming its DRB1∗09:01 restriction (Figures 3D and S4A). Furthermore, we employed the S_26–40_-DRB1∗09:01 tetramer for direct *ex vivo* assessment of antigen-specific CD4^+^ T cells in unexpanded PBMCs; tetramer-specific CD4^+^ T cells could be detectable, albeit at low baseline frequencies (Figure S4B). Additionally, most of these antigen-specific cells displayed a CD45RA^−^CCR7^−^ phenotype, identifying them as effector memory T cells (T_EM_), with a subset expressing the differentiation marker CD27 and the activation marker PD-1 (Figure S4C).

Collectively, we discovered four previously unrecognized pairs of CD4^+^ T cell immunodominant epitope-MHC-II restriction: DPB1∗05:01-restricted N_106–120_ (N22), N_261–275_ (N53), DRB1∗15:01-restricted S_126–140_ (S26), and DRB1∗09:01-restricted S_26–40_ (S6). Given the high prevalence of DPB1∗05:01, DRB1∗09:01, and DRB1∗15:01 in Han Chinese populations (Table S5), these epitopes may represent promising candidates for developing regionally tailored T cell vaccine designs.

### Longitudinal epitope-specific dynamics of SARS-CoV-2 T cell immunity

Previous studies have revealed that SARS-CoV-2-specific CD4^+^ and CD8^+^ T cells expand during the acute phase of infection (within the first 2–4 weeks post-symptom onset) and subsequently declined with an estimated half-life of 8 months based on bulk T cell response measurements to viral antigen pools.^22^^,^^23^^,^^24^ However, these studies lack the dynamics of SARS-CoV-2-specific T cells at single-epitope resolution, leaving critical gaps in understanding the long-term maintenance of T cell immunity. To address the question, we conducted longitudinal analyses of CD4^+^ and CD8^+^ T cell response dynamics at single-epitope resolution across two 10- to 12-month intervals: (1) spanning one month after booster inactivated vaccination and one month post-breakthrough infection, and (2) spanning one month and ten months after Omicron breakthrough infection.

**Figure 4:**
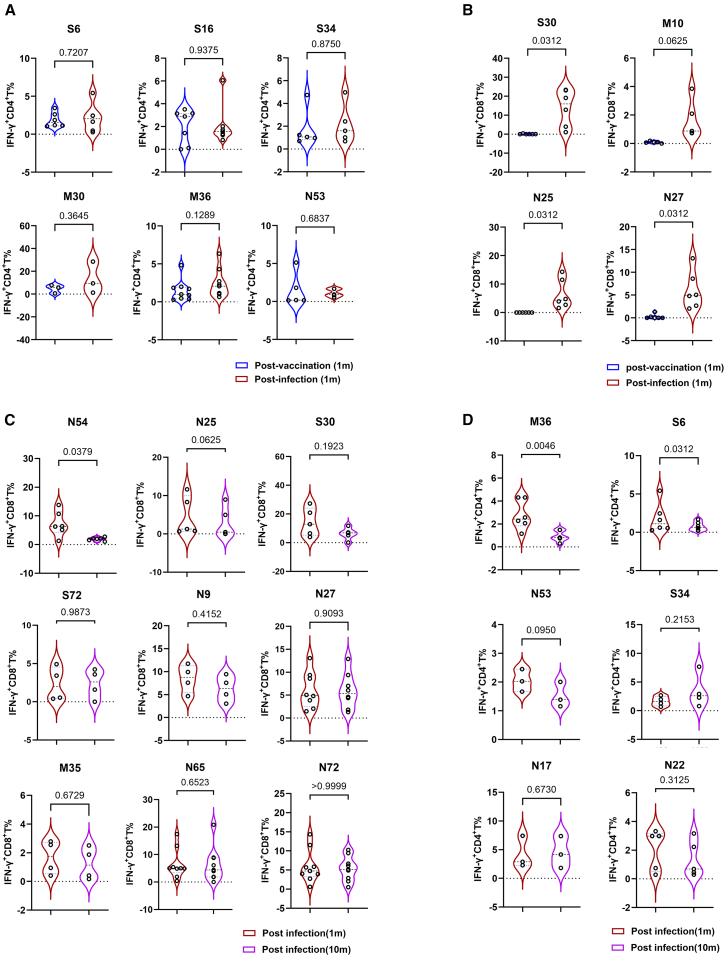
Longitudinal epitope-specific dynamics of SARS-CoV-2 T cell immunity (A and B) Flow cytometry detection of CD4^+^ T cell response (A) and CD8^+^ T cell response (B) to indicated epitope-containing peptides in S1, N, or M peptide pool-pre-expanded PBMCs of responders at one month after booster inactivated vaccination and one month post-breakthrough infection. (C and D) Flow cytometry detection of the responses of IFN-γ^+^ CD8^+^ T cells (C) and IFN-γ^+^ CD4^+^ T cells (D) to indicated epitope-containing peptides in S1, N, or M peptide pool-pre-expanded PBMCs from convalescent responders with breakthrough infection at 1 and 10 months post-infection. Paired *t* tests or Wilcoxon tests for normally or non-normally distributed data, respectively in (A)–(D). Data represent independent biological replicates in (A)–(D). Violin plots display quartile values or means.

Epitope-specific CD4^+^ T cell responses showed no significant change between one month post-booster inactivated vaccination and one month post-breakthrough infection (Figure 4A). Since we previously compared T cell responses to the S1 peptide pools before and after booster vaccination at one and five months using unexpanded PBMCs, we found that the booster vaccination induced a strong and persistent CD4^+^ T cell response that lasted for at least five months (Figure S5A and Table S6). So, we speculated that inactivated vaccination elicited pre-existing CD4^+^ T cells. In contrast, epitope-specific CD8^+^ T cell responses were detectable only following breakthrough infection, but not in individuals one month after booster inactivated vaccination (Figure 4B). Similarly, we evaluated the dynamics of CD8^+^ T cell responses before and after a booster vaccination. Our results show no significant increase in S1-specific CD8^+^ T cell responses at one month post-booster (Figure S5B). Taken together, these findings indicated that the inactivated vaccine platform was a robust inducer of CD4^+^ T cells but elicited weak CD8^+^ T cell immunity. Longitudinal monitoring revealed that the majority of epitope-specific CD8^+^ T cells (except for N54-specific T cells) maintained stable frequencies over time (Figure 4C). Similarly, progressive declines were observed in only two epitope-specific CD4^+^ T cell populations (S6 and M36) (Figure 4D). So, the temporal dynamics of memory T cells is epitope-specific after SARS-CoV-2 infection.

### Mutations at epitope core site impair HLA-mediated antigen presentation

**Figure 5:**
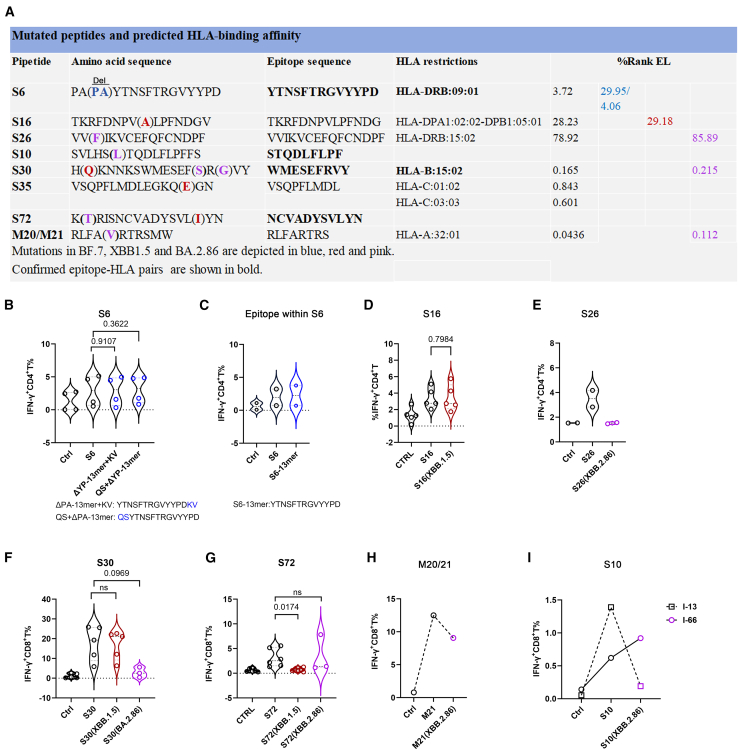
Mutations at epitope core site of Omicron subvariants XBB.1.5, BA.2.86, and BF.7 mediate immune evasion from T cell immunity (A) Sequence alignment highlighting epitope mutations in SARS-CoV-2 S1 and M protein across Omicron subvariants (XBB.1.5, BA.2.86, and BF.7) and corresponding HLA-binding affinity by NetMHCpan 4.1 (HLA-I) or NetMHCIIpan 4.0 (HLA-II). (B and C) CD4^+^ T cell responses to the original and mutated S6 peptides (B) and epitopes within S6 (C) of S1 peptide pool-pre-expanded PBMCs from corresponding responders. (D and E) CD4^+^ T cell responses to the original and mutated S16 peptides (D) and S26 peptides (E) of S1 peptide pool-pre-expanded PBMCs from corresponding responders. (F–I) CD8^+^ T cell responses of the original and mutated S30 (F), S72 (G), M20/21 (H), and S10 (I) peptides of S1 or M peptide pool-pre-expanded PBMCs from corresponding responders. Paired *t* tests or Wilcoxon tests for normally or non-normally distributed data, respectively, in (B), (D), (F), and (G). Data represent independent biological replicates in (B)–(G) and one of two technical replicates using PBMCs from the indicated donors in (H) and (I). Violin plots display quartile values. PBMCs, peripheral blood mononuclear cells.

While bulk T cell assays have not identified widespread immune evasion by SARS-CoV-2 variants, emerging evidence suggests that mutations in specific epitopes can confer partial immune evasion.^25^^,^^26^^,^^27^^,^^28^^,^^29^^,^^30^ These findings underscore the necessity of epitope-resolved analyses for the evaluation of T cell immunity against viral evolution. To systematically assess immune evasion, we compared S1, N, and M protein sequences between the ancestral strain and Omicron subvariants (XBB.1.5, BA.2.8.6, and BF.7), identifying mutations in the epitope-containing peptides S16, S26, S30, S72, and M21 (Figure 5A).

For CD4^+^ T cell recognition, we compared T cell responses against S6, S16, and S26, which harbor mutations present in Omicron subvariants. The BF.7 strain carries a consecutive two amine acid deletion in the S6 peptide compared with the ancestral strain. Nevertheless, the two mutated S6 peptides (ΔPA-13mer+KV: YTNSFTRGVYYPDKV; QS+ΔPA-13-mer: QSYTNSFTRGVYYPD) elicited CD4^+^ T cell responses at levels comparable to those induced by the original S6 peptide, excluding the possibility of YP within the core epitope residues of S6 peptide (Figure 5B). Furthermore, we assessed CD4^+^ T cell responses by the S6-13-mer peptide, which exhibited similar CD4^+^ T cell-stimulating activity to that by the original S6 peptide, confirming S6-13-mer as an epitope within S6 peptide (Figure 5C). XBB.1.5 subvariant carries one mutation in S16 epitope, but such mutation had no effect on CD4^+^ T cell responses (Figure 5D). By contrast, BA.2.86 subvariant has one mutation within S26, which significantly impaired epitope-specific CD4^+^ T cell response (Figure 5E).

For CD8^+^ T cell recognition, we evaluated CD8^+^ T cell responses to S10, S30, S72, and M20/21 epitopes carrying Omicron mutations. The S30 peptide acquired three mutations: two within its core epitope (BA.2.86 lineage, position 157–158) and one in a non-core region (XBB.1.5 lineage, position 146) (Figure 5A). Consistent with computational predictions, the mutated S30 peptide in XBB.1.5 still elicited comparable CD8^+^ T cell responses as the ancestral peptide. In contrast, the mutated S30 harbored by BA.2.86 failed to activate CD8^+^ T cell responses, despite *in silico* predictions indicating preserved B∗15:02 binding affinity (Figures 5A and 5F). The XBB.1.5 mutation in the S72 core epitope (position 368) completely abolished CD8^+^ T cell responses, whereas the BA.2.86 mutation (position 356) outside the core had no effect (Figure 5G). The mutation in M20/21 (BA.2.86 lineage) did not impair CD8^+^ T cell responses (Figure 5H). Additionally, the BA.2.86 mutation in S10 epitope impaired CD8^+^ T cell response in one responder but not in another (Figure 5I), potentially due to differential effects on binding affinity across its multiple HLA class I restrictions (Table S2).

Collectively, we identified mutations in the S30 (F157S and R158G), S72 (L368I), and S10 (S50L) epitope mediating CD8^+^ T cell immune evasion, as well as a mutation in the S26 (V127F) epitope mediating CD4^+^ T cell immune evasion, across SARS-CoV-2 Omicron subvariants.

## Discussion

The extensive diversity of T cell epitopes, combined with the high polymorphism of the HLA system, constitutes a fundamental immunological barrier enabling humans to combat viral infections and mitigate pandemic spread. Systematic identification of dominant antigenic epitopes and their HLA restriction profiles in target populations provides an essential foundation for deciphering antiviral T cell response mechanisms. In this study, we validated one T cell epitope screening approach using hierarchical peptide pool arrays. Future work will focus on optimizing *in vitro* expansion protocols for antigen-specific T cells to enhance detection sensitivity and throughput, thereby facilitating high-efficiency screening of functional T cell clones.

Our study identified multiple immunodominant CD4^+^ T cell epitopes and revealed notable differences in the persistence of T cell responses against them. Interestingly, only two epitopes—S6 and M36—showed progressive declines over time. A likely explanation involves the functional heterogeneity of CD4^+^ T helper (Th) cells. Upon activation, naive T cells can differentiate into distinct subsets such as Th1, Th17, or intermediate phenotypes like Th1/17, each with different lifespans and stability *in vivo*.^31^ We hypothesize that the S6 and M36 epitopes may preferentially drive the differentiation of Th subsets that are inherently short-lived or more prone to activation-induced exhaustion, unlike those triggered by other, more stable epitopes.^32^ To better understand the distinct dynamics of epitope-specific T cell subsets, comprehensive functional profiling is necessary.

The limited activation of CD8^+^ T cells following a booster dose of inactivated vaccines can be attributed to their exogenous antigen nature, which necessitates cross-presentation for CD8^+^ T cell activation. Inactive vaccine-induced CD8^+^ T cell epitopes may exhibit distinct epitope-specific and functional characteristics compared to those elicited by endogenous viral replication during natural infection. This hypothesis warrants a systematic investigation of epitope specificity in vaccine-primed CD8^+^ T cells, particularly through comparative analyses across three antigen presentation paradigms: (1) breakthrough infections (mainly endogenous viral antigen processing), (2) exogenous antigen vaccines (e.g., inactivated vaccines), and (3) endogenous antigen vaccines (e.g., mRNA or adenoviral vector platforms). Such comparative epitope mapping could provide mechanistic insights into how antigen presentation routes shape the functional avidity and clonal diversity of CD8^+^ T cell populations. Importantly, our findings suggest that heterologous prime-boost regimens combining exogenous and endogenous vaccine modalities may synergistically broaden the pre-existing memory T cell repertoire, simultaneously enhancing both CD4^+^ T cell coordination and CD8^+^ cytotoxic T cell coverage.^33^^,^^34^^,^^35^^,^^36^^,^^37^^,^^38^ This combinatorial approach holds potential to overcome single-platform vaccination limitations, thereby establishing multi-layered T cell immunity capable of recognizing diverse viral variants through complementary epitope-targeting strategies.

Effective SARS-CoV-2-specific CD4^+^ T cells are predominantly Th1 and Tfh subsets: Th1 cells support effector CD8^+^ T cell generation, while Tfh cells drive neutralizing antibody responses, collectively contributing to viral clearance and propagation suppression.^39^^,^^40^ The S34 peptide demonstrates multifaceted immunogenic potential, building upon Peng et al.’s original identification of its dual CD4^+^/CD8^+^ T cell epitope.^10^ Mechanistically, Mudd et al. demonstrated that BNT162b2 mRNA vaccination establishes persistent S34-DPB1∗04-specific Tfh cells within the draining lymph nodes, a process essential for germinal center-mediated B cell maturation.^5^ Our findings expand the functional diversity of epitope-specific T cells by demonstrating that inactivated vaccine recipients develop robust S34-DPB1∗04-directed Th1 responses. This conserved immunodominance of S34 across divergent vaccine platforms (e.g., mRNA vs. inactivated) highlights its unique capacity to bridge cellular and humoral immunity, with the broad relevance of epitope-specific responses across distinct vaccine platforms.

It is widely recognized that specific HLA alleles exhibit strong associations with strong susceptibility to human diseases, particularly autoimmune inflammatory disorders. Identification of SARS-CoV-2-specific epitope-HLA pairs may provide valuable insights into the mechanistic relationships between HLA polymorphisms and disease pathogenesis. Among the study participants, a subset carries B∗51, the first identified and most strongly associated genetic factor for the Behçet’s disease (BD).^41^^,^^42^ BD is a chronic inflammatory disorder with unknown etiology, characterized by inflammation in oral aphthous ulcers, genital ulcers, uveitis, and skin. Excessive production of pro-inflammatory cytokines (predominantly Th1 type) appears to contribute to the autoimmune pathogenesis in the disease. Endothelial activation and injury, along with their consequent occlusive vasculopathy, may additionally contribute to tissue damage.^43^ A previous study revealed that the association between MHC-I and BD was localized to six key positions near the peptide-binding groove. These sites are crucial for determining peptide-MHC-I binding affinity and subsequent recognition of MHC-I molecules by the receptors on natural killer (NK) cells and T cells.^44^ MHC-I molecules contribute to BD pathogenesis possibly by activating cytotoxic NK cells or CD8^+^ cytotoxic T lymphocytes (CTLs) that are initially primed by infection. On the other hand, in addition to independent genetic factors (e.g., PSORS1C1 and Cw∗1602), HLA linkage disequilibrium has also been implicated in the pathogenesis of BD. Specifically, the HLA-B/MICA region and the region between HLA-F and HLA-A have each been independently associated with BD.^42^^,^^44^ Notably, an earlier study revealed the significant disequilibrium of B51∗ and C14∗ in BD patients, with only B51∗, but not C14∗, identified as a potential pathogenic factor.^45^ Therefore, whether the COVID-19 pandemic influences the incidence rate of BD onset in HLA-B∗51 populations, including those with B∗51 disequilibrium, requires further investigation.

Above all, a comprehensive understanding of SARS-CoV-2-specific T cells requires integration of both global antigen-level and epitope-resolved approaches. This combined strategy enables precise dissection of how mutation-driven immune evasion impacts bulk T cell memory, while simultaneously interrogating *in vivo* memory maintenance dynamics and disease severity-associated T cell response patterns. In addition, given that spike (S) protein mutations frequently undermine T cell recognition, next-generation vaccines should prioritize conserved immunodominant epitopes residing in non-spike antigens—such as the N protein’s structural domains—to establish broadly protective T cell immunity against viral mutations. Inactivated vaccines elicit broad-spectrum CD4^+^ T cell responses due to their inclusion of a wider array of structural proteins compared with other vaccine platforms. From both the magnitude and breadth perspectives, heterologous immunization regimens combining inactivated vaccines with intracellular antigen-targeting vaccines (e.g., mRNA or viral vector-based vaccines) could offer a viable strategy to mitigate future infectious disease threats.

### Limitations of the study

It is important to note that our pre-expansion screening method has limitations. The potential overgrowth of highly proliferative clones during culture could consequently prevent complete capture of the antigen-specific T cell repertoire. Integrating this method with direct detection techniques in future research would thereby provide a more unbiased and comprehensive analysis of T cell immune responses. We screened the structural protein epitopes not covering S2 protein and non-structural accessory proteins, which also elicit robust T cell responses. Further studies are needed to focus on the epitopes in non-structural proteins, using the same PBMCs. In addition, we limited our analysis to Th1 cytokine production and did not study other CD4^+^ T cell subsets such as Tfh. Furthermore, all the volunteers of our study were categorized as having asymptomatic or mild disease. Further studies are needed to examine the relationship between these epitope-HLA pairs with the disease severity by detecting PBMC samples of volunteers with different disease severity. In addition, while volunteer sex information was recorded, the correlation between sex/gender and SARS-CoV-2 T cell epitope responses was not analyzed due to sample size constraints.

## Resource availability

### Lead contact

Requests for further information and resources should be directed to and will be fulfilled by the lead contact, Shuxun Liu (liusx@immunol.org).

### Materials availability

S_152-160_-B∗15:02 dextramer and S_26-40_-DRB1∗09:01 tetramer can be obtained upon reasonable request from the corresponding authors.

### Data and code availability

This paper does not report original code and datasets. The data files supporting this study can be obtained upon reasonable request from the corresponding authors.

## Acknowledgments

We thank professor Xuetao Cao for helpful discussion, Xiongfei Xu for his assistance in HLA epitope analysis, as well as Xinyue Deng, Tingting Fang, and Jia Xu for their technical support with blood sample collection. This work was supported by grants from the Emergency Key Program of Guangzhou Laboratory (EKPG21-30-3), 10.13039/501100001809National Natural Science Foundation of China (32370945, 91942304, and 92269204) and Young Scientist Project of the 10.13039/501100012166National Key Research and Development Program of China (2023YFA1801400).

## Author contributions

Conceptualization, S.L., Z.L., and Y.Y.; data curation, formal analysis, and visualization, Z.L. and S.L.; investigation, Z.L, M.C., T.H., J.W., J.D., and X.C.; funding acquisition, Z.L., S.L., and J.H.; resources, S.L., Z.W., and Y.Y.; project administration and supervision, S.L. and Y.Y.; writing – original draft, Z.L. and S.L.; writing – review & editing, Z.L., S.L., J.W., and Q.S.

## Declaration of interests

The authors declare no competing interests. Naval Medical University and Guangzhou Laboratory have filed for patent protection regarding the identification of specific epitopes.

## STAR★Methods

### Key resources table

**Table:** 

REAGENT or RESOURCE	SOURCE	IDENTIFIER
**Antibodies**		

Anti-human CD3-BUV805	BD Biosciences	Cat# 612893; RRID:AB_2870181
Anti-human CD3-BUV737	BD Biosciences	Cat# 612752;RRID:AB_287008313
Anti-human CD3-BV605	BD Biosciences	Cat# 563219;RRID:AB_2738076
Anti-human CD8-APC	BD Biosciences	Cat# 561953;RRID:AB_10896290
Anti human CD8-APC-R700	BD Bioscience	Cat# 565165;RRID:AB_2744457
Anti-human CD8-AF488	BD Bioscience	Cat# 557696;RRID:AB_396805
Anti-human CD8-BUV395	BD Bioscience	Cat# 563795;RRID:AB_2722501
Anti-human CD4-V450	BD Bioscience	Cat# 561838;RRID:AB_10924599
Anti-human CD4-BV785	Biolegend	Cat# 300554;RRID:AB_2564382
Anti-human CD4-APC-Fire750	Biolegend	Cat# 344638;RRID:AB_2572097
Anti-human CD4-BUV661	BD Bioscience	Cat#612962;RRID:AB_2870238
Anti-human CD45RA-BB700	Biolegend	Cat# 742249;RRID:AB_2871441
Anti-human CD45RA-Super Bright™ 702	Invitrogen	Cat# 67045842;RRID:AB_2662460
Anti-human CXCR5-PE-Dazzle594	BD Bioscience	Cat# 356928;RRID:AB_2563689
Anti-human CD161-PE-Cy5	Biolegend	Cat# 339951;RRID:AB_2893471
Anti-human TCR Vα7.2-PE-Cy7	Biolegend	Cat# 351712;RRID:AB_2561994
Anti-human CCR7-PE-Cy5	Biolegend	Cat# 353272;RRID:AB_2904373
Anti-human-IFN-γ-PE	Biolegend	Cat# 506507;RRID:AB_315440
Anti-human-IFN-γ-BV421	Biolegend	Cat# 506538;RRID:AB_2801098
Anti-human-IFN-γ-BB700	BD Bioscience	Cat# 566394;RRID:AB_2744484
Anti-human CD69-PE-CF594	BD Bioscience	Cat# 562617;RRID:AB_2737680
Anti-human CD127-BV421	BD Bioscience	Cat# 562436;RRID:AB_11151911
Anti-human PD-1-BB515	BD Bioscience	Cat# 564494;RRID:AB_2738827
Anti-human CD27-BV650	BD Bioscience	Cat# 564894;RRID:AB_2739004
Anti-human CD28-BV605	Biolegend	Cat# 302968;RRID:AB_2800755
Anti-human GZMB-RB705	BD Bioscience	Cat# 570247;RRID:AB_3685609
Anti-human IL-2-APC	Biolegend	Cat# 500311;RRID:AB_315098
Anti-human TNF-α-PE-Cy7	BD Bioscience	Cat# 2131650;RRID:AB_1727578
Anti-HLA-DR	Biolegend	Cat# 307602;RRID:AB_314680
Anti-HLA-DP	Leinco Technologies	Cat# H260;RRID:AB_2737518

**Biological samples**		

PBMCs	This study	N/A

**Chemicals, peptides, and recombinant proteins**		

15mer-peptides spanning the S1 protein	GL Biochem Corporation	N/A (Custom)
15mer-peptides spanning the N protein	GL Biochem Corporation	N/A (Custom)
15mer-peptides spanning the M protein	GL Biochem Corporation	N/A (Custom)
Predicted epitopes within 15mer peptides	GL Biochem Corporation	N/A (Custom)
Mutation peptides	GL Biochem Corporation	N/A (Custom)
HLA-B∗15:02-WMESEFRVY-dextramer-APC	Immudex	N/A (Custom)
DRB09:01:02-PAYTNSFTRGVYYPD-Tetramer-PE	ProImmune	N/A (Custom)
Flex-T™ Biotin HLA-A∗11:01 SARS-CoV-2 Monomer UVX	Biolegend	280007
Streptavidin-PE	Biolegend	405203
Streptavidin-BV421	Biolegend	410505
Recombinant Human IL-2 Protein	SL PHARM	S20040020
RPMI Medium 1640	Gibco	11875-093
Fetal Bovine Serum	Gibco	10099-141
Fetal Bovine Serum	Biowest	S1580-500
BFA	Invitrogen	00-4506-51
Cytofix/Cytoperm™ Fixation/Permeabilization	BD Bioscience	554714
DMSO	Sangon Biotech	A100231
Ficoll density gradient centrifugation	Serumwerk Bernburg AG	04-03-9391/02
D-Biotin	Thermo fisher	B20656
20×PBS	Sangon Biotech	B458117-0500
Human Trustain FcX blocking reagent	Biolegend	422301
Zombie NIR™ fixable viability dye	Biolegend	423106

**Software and algorithms**		

GraphPad Prism v 10.2.1	GraphPad	https://www.graphpad.com/
FlowJo v 10.10.0	FlowJo	https://www.flowjo.com/
NetMHCpan-4.1		NetMHCpan 4.1 - DTU Health Tech - Bioinformatic Services
NetMHCIIpan-4.0		NetMHCIIpan 4.0 - DTU Health Tech - Bioinformatic Services

### Experimental model and study participant details

#### Human participants

In this longitudinal cohort study, we enrolled 385 individuals who received primary immunization with two doses of inactivated COVID-19 vaccines (CoronaVac [Sinovac] or BBIBP-CorV [Sinopharm]) by May 2021, followed by homologous booster vaccination in January 2022. PBMC samples were collected from these volunteers at 1-month pre-booster and 1- and 5-month post-booster vaccination. Most cohort participants later developed SARS-CoV-2 breakthrough infections with Omicron BA.5/BF.7 subvariants, confirmed by rapid antigen testing during the BA.5/BF.7-dominant wave (December 2022 to January 2023). PBMC samples were collected from 92 of these volunteers at 1-month and 10-month post-infection. Further analysis focused on 58 participants exhibiting robust T cell responses post-infection, with detailed infection dates and blood collection timelines provided in Table S1. To assess the T-cell response induced by the booster immunization, we analyzed a cohort of 57 participants who had received three doses of the inactivated vaccine. The vaccination schedule is detailed in Table S6 (some individuals overlapping with those in Table S1). This study was approved by the Institutional Review Boards of the First Affiliated Hospital of Guangzhou Medical University (2021-78) and the Naval Medical University. Written informed consent was obtained from all participants prior to enrollment.

### Method details

#### Peptide pool design and preparation

Peptide libraries encompassing wild-type SARS-CoV-2 (D614G) structural proteins (S1, N, M) were designed as 15-mer sequences with 10-amino-acid overlaps, comprising 136 peptides spanning S1 protein, 82 peptides for N protein, and 43 peptides for M protein. All synthetic peptides (HPLC purity >90%) were reconstituted in DMSO at 20 mM stock concentrations and systematically organized into hierarchical pools: full-protein pools, mesopools and minipools. Additionally, computationally predicted epitopes and mutation-specific peptides encompassing signature substitutions from emerging Omicron sublineages (XBB.1.5, BA.2.86 and BF.7) were synthesized and reconstituted as described above.

#### Preparation of PBMCs

PBMCs were isolated as described previously using Ficoll density gradient centrifugation and cryopreserved using FBS containing 7.5% dimethyl sulfoxide at -80°C overnight and then transferred to liquid nitrogen for further preservation.^46^

#### *In vitro* PBMC expansion and culture

Short-term SARS-CoV-2-specific T-cell lines were generated using a modified protocol.^47^ Cryopreserved PBMCs were thawed and seeded at 3×10^5^ cells/well in 96-well U-bottom plates with complete RPMI-1640 medium supplemented with 10% heat-inactivated FBS. Cells were pulsed with SARS-CoV-2 full-protein peptide pools (S1/N/M proteins, 250 μM per peptide) and incubated at 37°C with 5% CO_2_. Recombinant IL-2 (20U/mL) was added on day 3, followed by a 6-day expansion with medium replacement (50% volume exchange with fresh complete medium and 20U/mL IL-2) on day 6. Expanded cells were sub-cultured when needed on day 7. Expanded SARS-CoV-2-specific T-cell lines were either immediately for analysis or cryopreserved until subsequent assays.

#### T cell stimulation

Antigen-specific T cells were detected via intracellular cytokine staining under two conditions. For pre-expanded PBMCs, cells (3×10^5^/well) were restimulated with SARS-CoV-2 peptide mesopools or minipools (S1/N/M) or individual 15-mer peptides (250nM per peptide) for 1 hour, followed by 5-hour incubation with protein transport inhibitor cocktail containing brefeldin A (1:1000). In parallel, unexpanded cryopreserved PBMCs (3×10^5^/well) were stimulated with S1 peptide pools (250 nM) for 12 hours, with brefeldin A added for the final 4 hours. All stimulations were conducted in 96-well U-bottom plates at 37°C and 5% CO_2_. Subsequently, the cells were harvested for staining.

#### Intracellular cytokine staining assay

All staining procedures were performed in 1×PBS supplemented with 0.1% FBS. Following stimulation, cells were surface-stained with fluorescent-conjugated antibodies: BUV737 anti-CD3, APC/Fire 750 anti-CD4, and AF488 anti-CD8α. For the analysis of antigen-specific T cells in unexpanded PBMCs, the surface staining panel was supplemented with CD45RA, CCR7, and CD69, along with the Zombie NIR™ fixable viability dye. Following fixation/permeabilization with Cytofix/Cytoperm™, intracellular staining was performed using PE-conjugated anti-IFN-γ (clone B27) under optimized conditions: 16-hour incubation at 4°C or 2-hour at 25°C. Cells were washed with Perm/Wash™ Buffer and acquired within 24 hours on BD LSRFortessa™ X-20 or Sony ID7000™ spectral cytometers. Flow cytometry data were analyzed using FlowJo™ v10.10.0 .

#### Systematic high-throughput screening strategy for identifying T-cell epitope-containing peptides within SARS-CoV-2 structural proteins

We implemented a hierarchical deconvolution strategy (mesopools → minipools → individual peptides) for epitope mapping. The S1 and N, M protein libraries (136, 82, 43 peptides respectively) were partitioned into five mesopools (9-30 peptides per mesopool) representing distinct antigenic regions and, S1 and N peptides of mesopools subsequently were divided into six minipools containing 2-3 peptides to enable progressive epitope identification. Three iterative screening rounds were required for S1 and N protein epitope discovery, while the smaller membrane (M) protein library (43 peptides) necessitated only two rounds due to reduced combinatorial complexity (Figures 1A and S1). IFN-γ^+^CD4^+^/CD8^+^T-cell frequencies were quantified using multiparametric ICS assays as described above, with background responses normalized against DMSO-exposed controls. Antigen-specific responses were defined as a stimulation index (experiment/control ratio) exceeding 1.5-fold for CD8^+^T cell and 1.3-fold for CD4^+^T cell.

#### Integrated computational and experimental determination of MHC restriction

Computational prediction of T-cell epitope immunogenicity and MHC restriction profiles was conducted using NetMHCpan-4.1 (HLA class I) and NetMHCIIpan-4.0 (HLA class II) algorithms. Experimental validation of CD4^+^T cell epitope HLA restriction also utilized MHC class II blocking antibodies (anti-HLA-DR or anti-HLA-DP). Expanded PBMCs were pre-incubated with 10μg/mL anti-HLA-DR (clone L243) or anti-HLA-DP (clone B7/21) for 30 min at 37°C prior to peptide stimulation.

#### Identification of HLA restriction by allogeneic PBMCs

HLA restriction analysis was performed using an allogeneic PBMC co-culture system.^21^ Partially HLA-I-matched allogeneic PBMCs (3×10^4^cells/well) were pulsed with individual peptides (N_45-55_/N25/N_266-276_/N54) or DMSO vehicle control for 2 h at 37°C to generate peptide-loaded antigen-presenting cells (APCs). These peptide-loaded or control APCs were co-cultured with N peptide pool-pre-expanded PBMCs containing N-specific T cells (2×10^5^ cells/well) from responders for 1 h, followed by 5 h brefeldin A incubation. Surface staining (CD3/CD4/CD8) and intracellular IFN-γ detection were performed using ICS as described above.

#### Generation of N_361-369_/A∗11:01-specific fluorescent tetramers

Fluorescent tetramers specific for the N_361-369_ epitope presented by HLA-A∗11:01 were generated using Flex-T™ Biotin HLA-A∗11:01 monomers (Biolegend), conjugated with either streptavidin-PE (Biolegend) or streptavidin-BV421 (Biolegend), respectively, following the manufacturer's protocol. Briefly, 30 μL of monomer was mixed with 3.3 μL of the respective streptavidin conjugate and incubated on ice in the dark for 30 minutes. A blocking solution was prepared by combining 1.6 μL of 50 mM D-biotin (Thermo fisher) with 198.4 μL of PBS. Following the incubation, 2.4 μL of this blocking solution was added to stop the reaction. The mixture was then incubated at 4°C overnight to complete tetramer formation.

#### Tetramer or dextramer phenotyping

Epitope-specific T cells were identified using N_361-369_/A∗11:01 tetramers, S_152-160_-B∗15:02 dextramer (Immudex), and S_26-40_-DRB1∗09:01 tetramer (ProImmune). Briefly, PBMCs-either post-expansion (3×10^5^) or thawed cryopreserved cells (1×10^6^)-were first incubated with Human TruStain FcX to block Fc receptors and minimize nonspecific binding. Cells were then stained with the corresponding tetramer or dextramer for 15 minutes at room temperature, followed by surface staining with antibodies against CD3, CD4, and CD8. For memory subset characterization, an extended antibody panel (CD161, TCR Vα7.2, CCR7, CD45RA) was used to identify conventional memory CD8^+^T cells, while non-Tfh memory CD4^+^T cells were defined based on the expression of CXCR5, CCR7, and CD45RA. In a subset of samples, the phenotype of S_26-40_–Tetramer^+^CD4^+^T cells and S_152-160_–Dextramer^+^CD8^+^T cells was further analyzed using a panel including CD27-BV650, CD28-BV605, CCR7-PE-Cy5, and PD-1-BB515. All samples were acquired on either an LSRFortessa™ X-20 or ID7000 flow cytometer and analyzed using FlowJo v10. All antibodies and reagents are listed in the key resources table.

### Quantification and statistical analysis

#### Statistical analysis

Data and statistical analyses were performed in GraphPad Prism 10. Statistical details are provided in figure legends. Unless otherwise mentioned, P values <0.1 were considered significant.

## Supplemental information

Document S1. Figures S1–S5

Table S1. Demographic characteristics of individuals with SARS-CoV-2 Omicron BA.5/BF.7 breakthrough infections included in this study, related to STAR Methods

Table S2. The HLA genotypes and frequencies of IFN-γ^+^ CD8^+^ T cells among total CD8^+^ T cells in responding donors, related to Figure 1 and Table 1

Table S3. The HLA genotypes and frequencies of IFN-γ^+^ CD4^+^ T cells among total CD4^+^ T cells in responding donors, related to Figure 1 and Table 2

Table S4. HLA class IIA genotypes of S16 peptide-responding donors, related to Table 2

Table S5. HLA genotypic distribution patterns in the cohort of 54 participants, related to STAR Methods

Table S6. Demographic characteristics of the vaccinated cohort evaluated for booster-induced T cell responses, related to STAR Methods
