# Supplementary material for: Systematic profiling of SARS-CoV-2 structural protein-specific T cell epitopes in Omicron infections following inactivated vaccination
Source: iScience. 2026 Feb 3;29(3):114891. doi: 10.1016/j.isci.2026.114891 (PMC12927107; doi:10.1016/j.isci.2026.114891)
Supplement: Document S1. Figures S1–S5 [file mmc1.pdf]

iScience, Volume *29*

Supplemental information

Systematic profiling of SARS-CoV-2 structural protein-specific T cell epitopes in Omicron infections following inactivated vaccination

Zhiqing Li, Mengmeng Cui, Jian Wu, Tianju Hu, Junyan Dan, Xiaosu Chen, Qicong Shen, Jin Hou, Zhongfang Wang, Yizhi Yu, and Shuxun Liu

Supplemental information

Systematic Profiling of SARS-CoV-2 Structural Protein-Specific T Cell Epitopes in Omicron Infections Following Inactivated Vaccination

Zhiqing Li, Mengmeng Cui, Jian Wu, Tianju Hu, Junyan Dan, Xiaosu Chen, Qicong Shen, Jin Hou, Zhongfang Wang, Yizhi Yu, Shuxun Liu

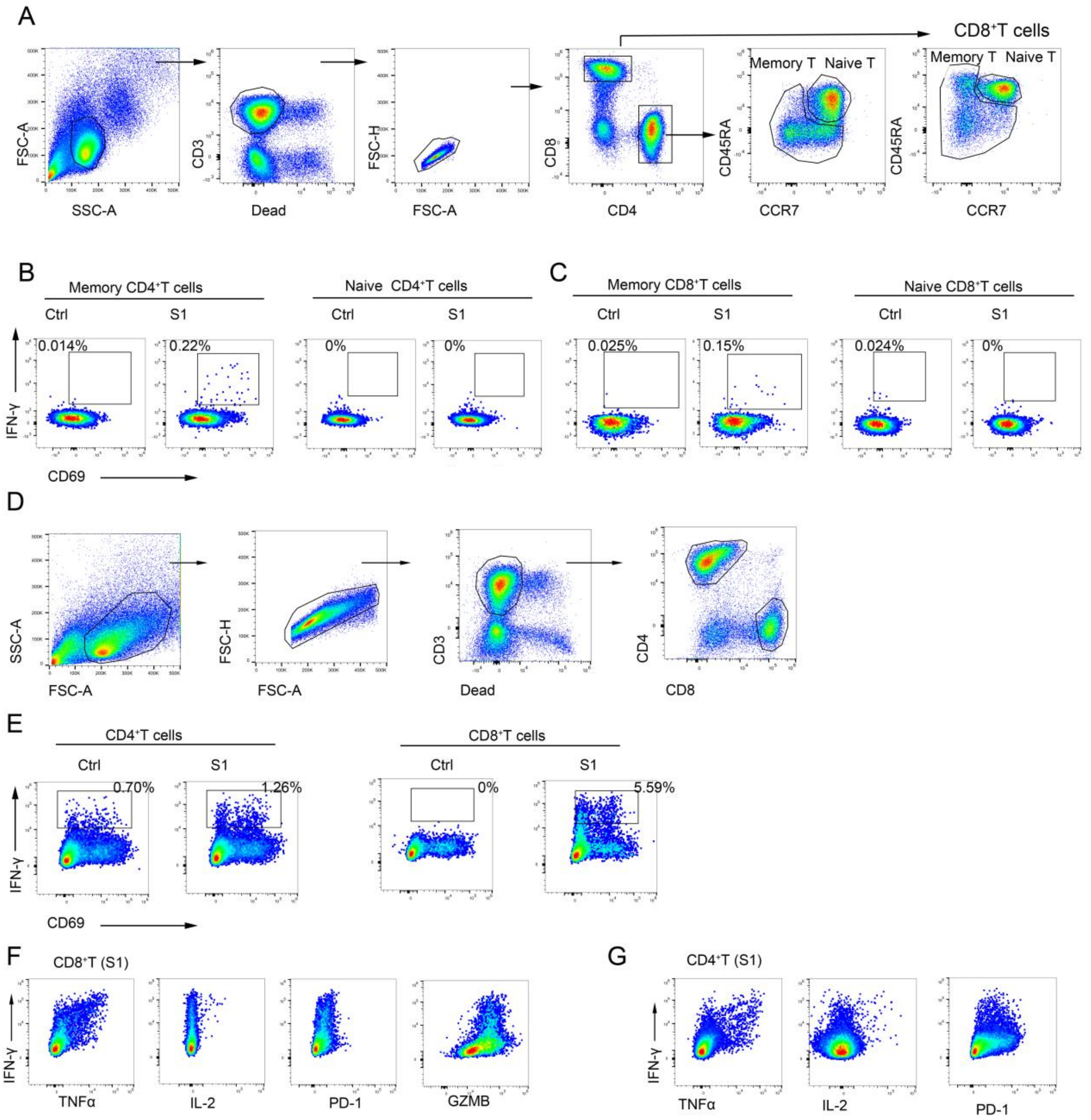

Figure S1. S1-specific T-cell responses in convalescent individual one month after breakthrough infection.

(A) Gating strategy for identifying naive and memory $CD4^+$ and $CD8^+$T-cell subsets in non expanded PBMCs.

(B, C) Representative flow cytometry plots of IFN-$\gamma^+$$CD69^+$ cells among (B) $CD4^+$and (C) $CD8^+$T cells following 16-hour stimulation with the S1 peptide pool or DMSO control in unexpanded PBMCs.

(D)Gating strategy for $CD4^+$ and $CD8^+$T cells in expanded PBMCs.

(E) Representative flow cytometry plots of IFN-$\gamma^+$$CD69^+$cells in $CD4^+$and $CD8^+$T cells after 5h stimulation with S1 peptide pool or DMSO control in expanded PBMCs.

(F-G) Cytokine production profiles and phenotypic characterization of (F) S1-specific $CD8^+$T cells and (G) S1-specific $CD4^+$T cells from expanded PBMCs after 5-hour peptide stimulation.

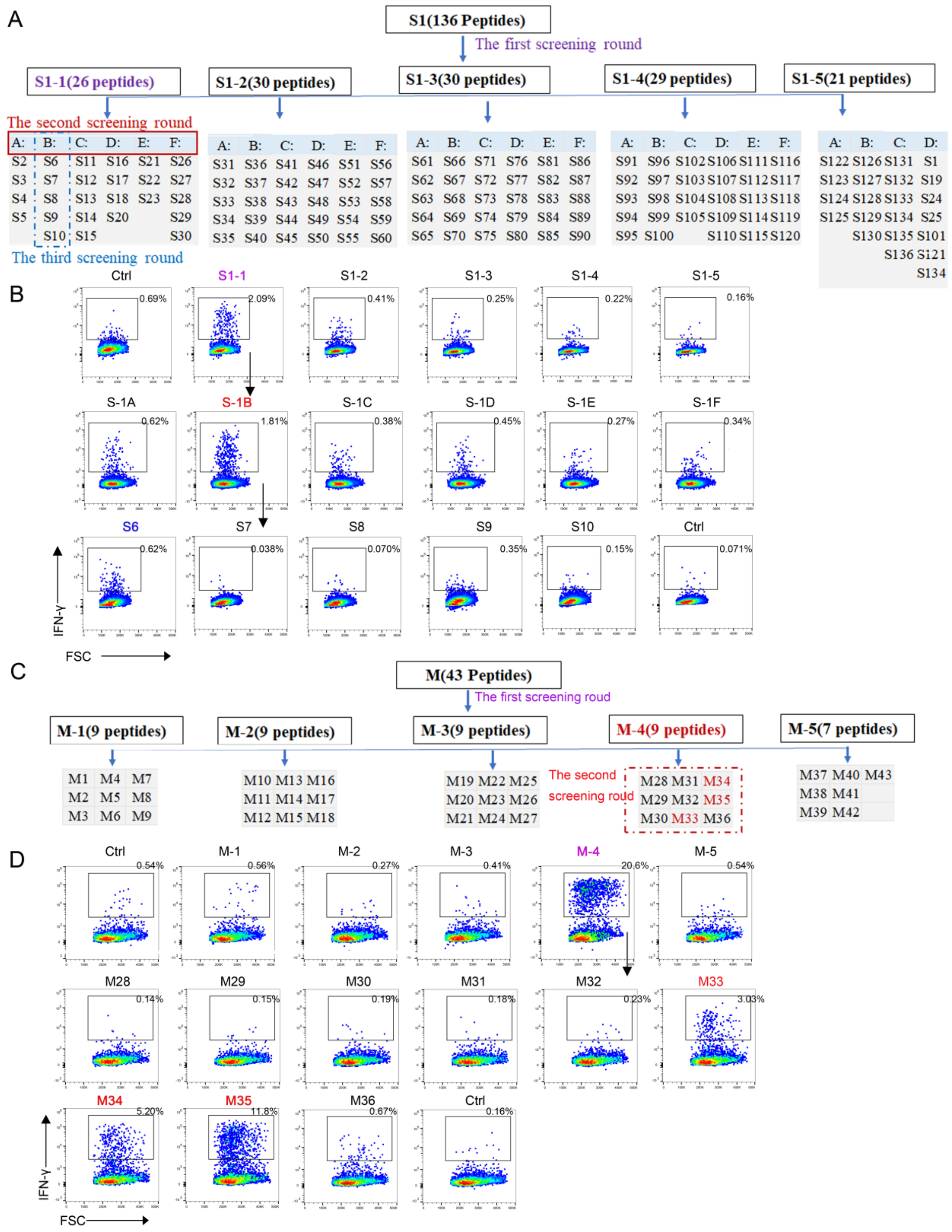

Figure S2. Identification of T cell epitope-containing peptides within S1 and M proteins, related to Figure 1.

(A) Screening strategy to identify T cell epitope-containing peptides in S1 protein.

(B) Flow cytometry plots showing the identification of the S6 peptide containing CD4^{+}T cell-epitope by three rounds of screening.

(C) Screening strategy to identify T cell epitope-containing peptides in M protein.

(D) Flow cytometry plots showing the identification of the M33, M34 and M35 peptides containing CD8^{+}T cell-epitope by two rounds of screening.

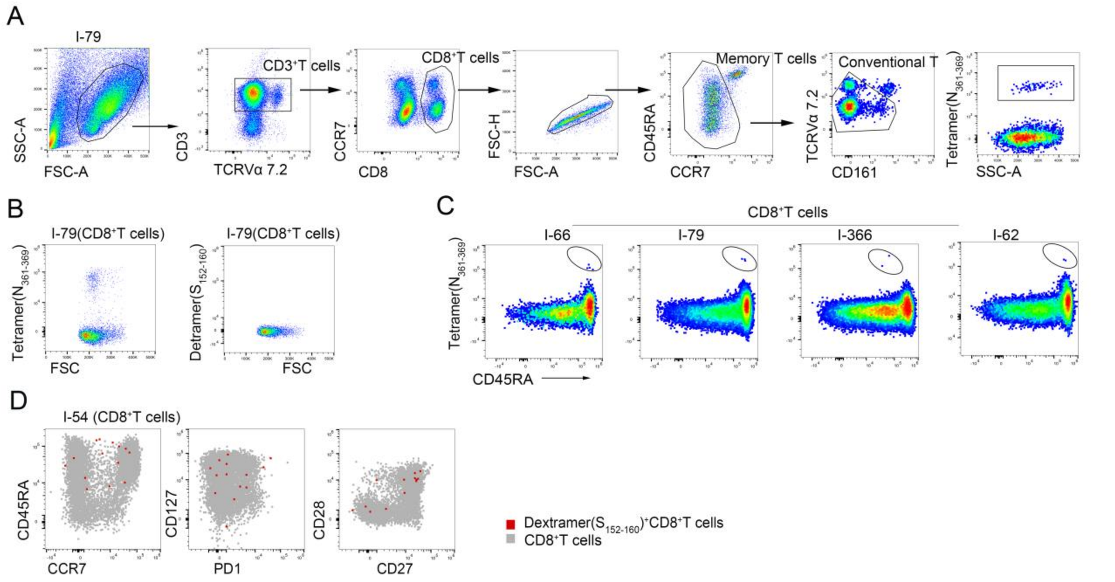

Figure S3. Identification of $N_{361\text{-}369}$-specific CD8$^+$T cells and $S_{152\text{-}160}$- specific CD8$^+$ T cells in PBMCs, related to Figure 2.

A. Gating strategy for the identification of $N_{361\text{-}369}$ tetramer$^+$ cells within memory conventional CD8$^+$T cells in Fig. 2J;

B. Flow cytometry measuring the $N_{361\text{-}379}$-specific T cells using the $N_{361\text{-}379}$ tetramer and the $S_{152\text{-}160}$ dextramer (as control) using the pre-expanded PBMCs by the N peptide pool from the donor I-79.

C. Flow cytometry detection of $N_{361\text{-}369}$-specific CD8$^+$T cells in thawed cryopreserved PBMCs without prior expansion from indicated responders using $N_{361\text{-}369}$ tetramer.

D. Flow cytometry measuring the phenotypes of the $S_{152\text{-}160}$-specific CD8$^+$T cells in thawed cryopreserved PBMCs without prior expansion from the donor I-54.

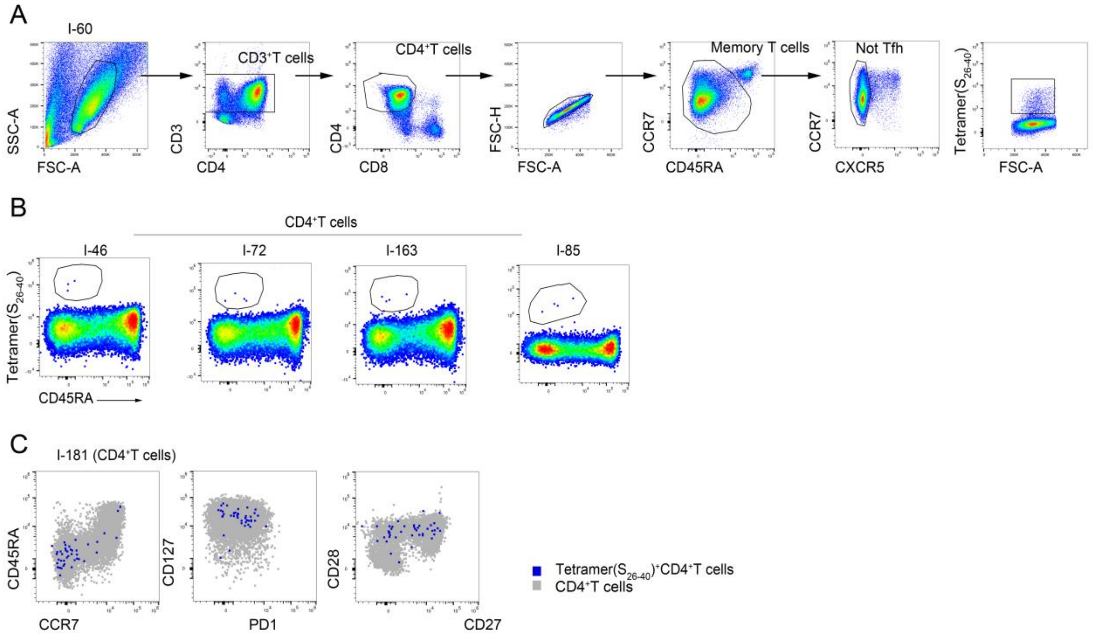

Figure S4. Identification of S_{26-40}-specific $CD4^+T$ cells in expanded and unexpanded PBMCs, related to Figure 3.

A. Gating strategy for the identification of S_{26-40} tetramer-binding non-Tfh memory $CD4^+T$ cells in Fig. 3D.

B. Flow cytometry detection of S_{26-40}-specific $CD4^+T$ cells in thawed cryopreserved PBMCs without prior expansion from indicated responders using S_{26-40}-DRB1*09:01 tetramer.

C. Flow cytometry measuring the phenotypes of the S_{26-40}-specific $CD4^+T$ cells in thawed cryopreserved PBMCs without prior expansion from the donor I-181.

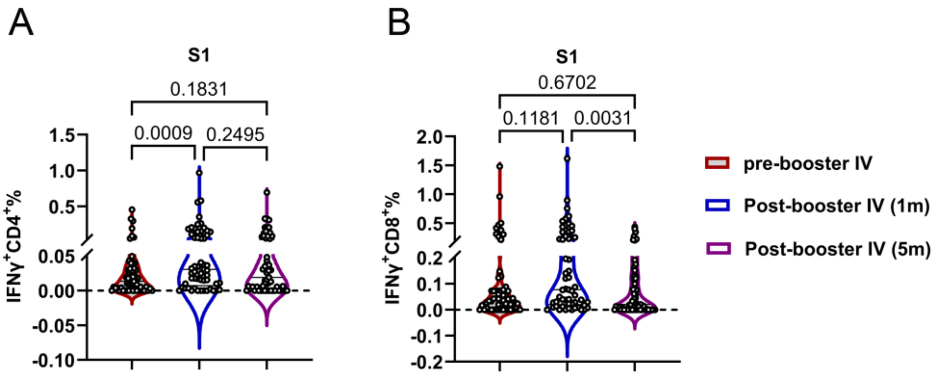

Figure S5. Longitudinal analysis of S1-specific T-cell responses pre- and post-booster vaccination, related to Figure 4.

$CD4^+$T(A) and $CD8^+$T cell(B) response to the S1 peptide pool by stimulating non-expanded PBMCs for 16h from donors at one and five months before and after receiving an inactive booster vaccination.
